# Nano-shaping of chiral photons

**DOI:** 10.1515/nanoph-2022-0779

**Published:** 2023-05-16

**Authors:** Yuji Sunaba, Masaki Ide, Ryo Takei, Kyosuke Sakai, Christophe Pin, Keiji Sasaki

**Affiliations:** Research Institute for Electronic Science, Hokkaido University, Sapporo, Hokkaido, Japan

**Keywords:** Laguerre–Gaussian mode, localized surface plasmon, nano-vortex, optically forbidden electronic transition, plasmonic nanoantenna

## Abstract

Localized surface plasmon polaritons can confine the optical field to a single-nanometer-scale area, strongly enhancing the interaction between photons and molecules. Theoretically, the ultimate enhancement might be achieved by reducing the “photon size” to the molecular extinction cross-section. In addition, desired control of electronic transitions in molecules can be realized if the “photon shape” can be manipulated on a single-nanometer scale. By matching the photon shape with that of the molecular electron wavefunction, optically forbidden transitions can be induced efficiently and selectively, enabling various unconventional photoreactions. Here, we demonstrate the possibility of forming single-nanometer-scale, highly intense fields of optical vortices using designed plasmonic nanostructures. The orbital and spin angular momenta provided by a Laguerre–Gaussian beam are selectively transferred to the localized plasmons of a metal multimer structure and then confined into a nanogap. This plasmonic nano-vortex field is expected to fit the molecular electron orbital shape and spin with the corresponding angular momenta.

## 1 Introduction

Laguerre–Gaussian (LG) beams, also called optical vortex beams, possess a doughnut intensity profiles and a helical wavefront. Since the unveiling by L. Allen et al. of the quantized, intrinsic nature of the orbital angular momentum carried by the helical wavefront of LG beams, the study of orbital angular momentum (OAM) and spin angular momentum (SAM) carried by photons have motivated numerous research works [1]. Especially, understanding how the light’s OAM and SAM couple to each other and how angular momentum exchange occurs in light–matter interactions is not only of fundamental interest but has also found applications in a wide range of fields. For instance, it was shown that helically structured light carrying intrinsic OAM can drive the orbital motion of microparticles [2], while elliptically polarized light carrying SAM rather induces the spinning motion of absorbing or birefringent particles [3–5]. Helically structured light is also used for super-resolution microscopy employing stimulated emission depletion (STED) [6], laser ablation for twisted metal structure fabrication [7, 8], and quantum information processing using multidimensional quantum states [9].

LG beams can be generated from a Gaussian (G) beam using a spiral phase plate or spatial light modulator forming azimuthally variant phase shifts [10]. Recently, it was demonstrated that plasmonic structures with chiral shapes could function as beam profile converters producing optical vortex fields [11–13]. Photons receive angular momentum from chirally structured materials. In contrast, multipolar plasmons of metal nanodisks with no chirality can be selectively excited by circularly polarized (CP) optical vortex beams [14–18]. In other words, OAM and SAM are transferred from LG-mode photons to localized plasmons.

Optically forbidden (OF) transitions, such as quadrupolar, hexapolar, and other multipolar excitations in molecules, can be freely induced by realizing the efficient transfer of the angular momentum from the optical vortex to molecular nanomaterials. This breakdown of the selection rules will dramatically improve the controllability and diversity of the photoreactions and photo processes. Recently, Schmiegelow et al. demonstrated that the quadrupolar transition of a ^40^Ca^+^ ion could be excited by an LG beam [19]. The quadrupolar interaction strength of the laser-trapped, cooled ^40^Ca^+^ ion was much larger than that of normal molecules. However, the angular momentum coupling between helically structured light and molecules is negligibly weak, considering the huge mismatch in the spatial extents of the photonic LG mode and molecular electron wavefunction. The diffraction limit restricts the minimum diameter of the optical vortex beam to the sub-micrometer scale. In contrast, the spread of the molecular orbital is on the nanometer or sub-nanometer scale. Recently, Heeres et al. experimentally demonstrated that a multi-antenna structure could induce vortex fields [20]. However, the relationship between the structure and field profile needs further clarification. Furthermore, their confined fields were on the hundred-nanometer scale. Therefore, the individual molecules are insensitive to the spatially variant phase shift of the optical vortex. In addition, the plasmonic near-field interaction, enhancing the localized field, hardly occurs in the hundreds of nanometer-sized structures [21] because the dipole–dipole interaction strength is inversely proportional to the distance cube.

**Figure 1:**
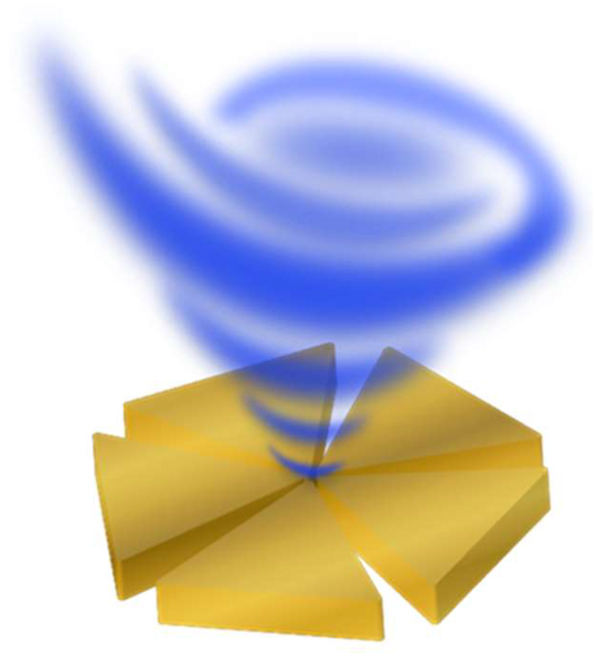
Nano-shaping of photons. Conceptual illustration of plasmonic nano-vortex fields formed by metal multimer structures, where the orbital angular momentum (OAM) and spin angular momentum (SAM) are transferred from Laguerre–Gaussian (LG)-mode beams to localized plasmons and then confined into nanometer-sized gaps.

This study proposes a novel method for forming single-nanometer-scale localized fields of optical vortices, drastically reducing the size mismatch in the helical profiles of photons and molecules. We employ gap-mode localized plasmons that produce strongly confined fields known as plasmonic hot spots. For instance, a plasmonic dimer, comprising two metal nanoparticles separated by a single-nanometer-scale gap, induces strong near-field interaction between the longitudinal electric waves of plasmonic resonances in the two particles, causing extreme localization effects and large field enhancements. The enormous spatial gradient of the electric field provided by the hot spot deviates from the long-wavelength approximation, i.e. the field amplitude and phase cannot be considered constant within a molecule volume, such that the molecule slightly transits to the OF states [22–24]. However, the original optically allowed (OA) transitions dominate molecular excitation because the field gradient is a first-order perturbation against the constant field approximation. Higher-order structural matching between photon modes and molecular orbitals is indispensable for controllable switching between OA and OF transitions. Here, we theoretically demonstrate that a tailored plasmonic multimer surrounding a single-nanometer-scale gap localizes the LG-mode field into the gap space by conserving high-order OAM and SAM (Figure 1). The relationship between the degrees of freedom in the multimer structures and transferable angular momenta is also discussed.

## 2 Results

**Figure 2:**
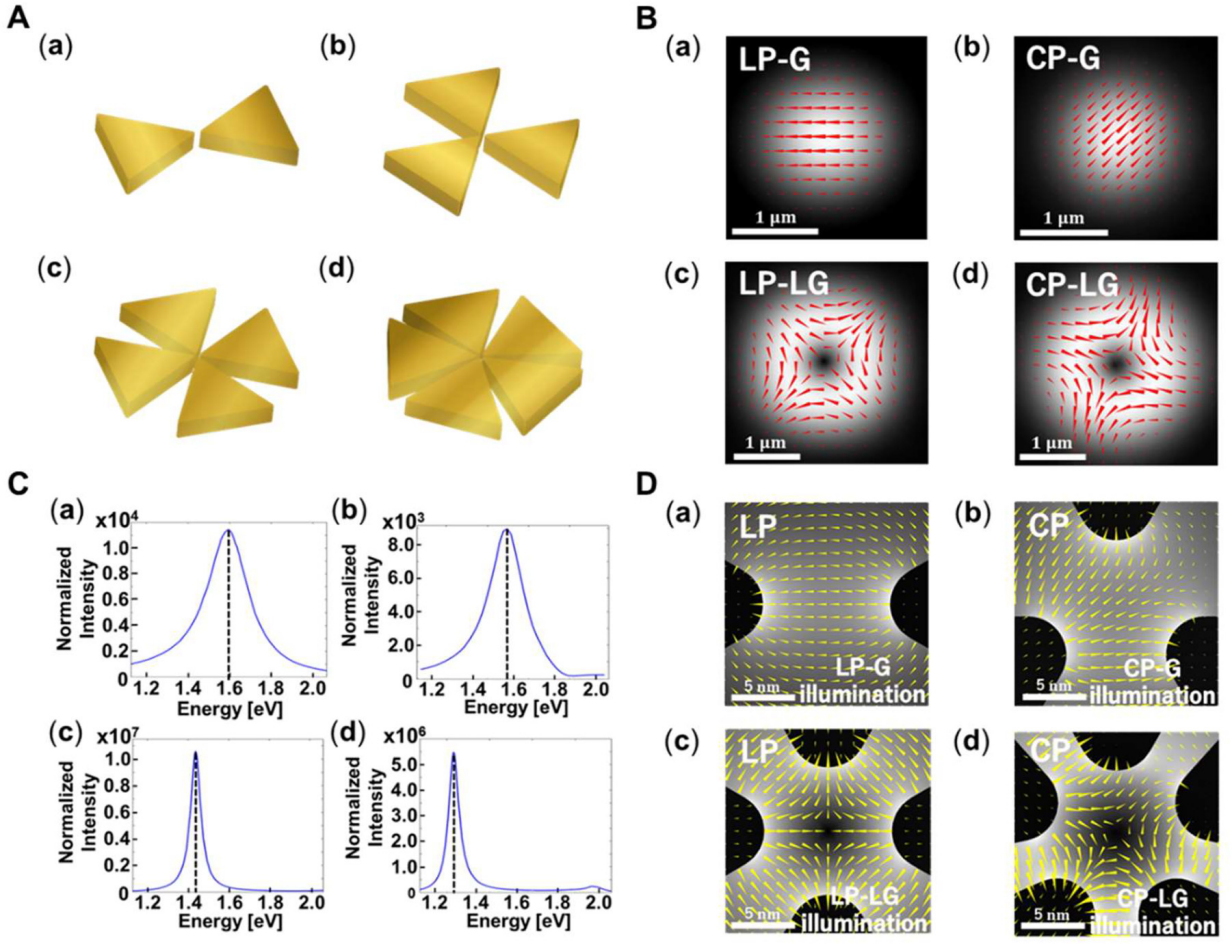
Optical vortex fields in nano-gaps of plasmonic multimer structures. (A) Designed nanostructures. (a) Dimer, (b) trimer, (c) tetramer, and (d) pentamer are composed of gold triangle blocks with 150-nm sides, 5-nm corner curvatures, and 30-nm thicknesses. The gap size is 10 nm. The surrounding medium is air. (B) Illumination beam profiles (average intensity and instantaneous electric field). (a) Linearly polarized Gaussian beam LP-G (*l* = 0, *s* = 0), (b) circularly polarized Gaussian beam CP-G (*l* = 0, *s* = 1), (c) linearly polarized Laguerre–Gaussian beam LP-LG (*l* = 0, *s* = ±1), and (d) circularly polarized Laguerre–Gaussian beam CP-LG (*l* = 1, *s* = 1), that are irradiated on the multimer structures (Aa–Ad), respectively. Red arrows represent electric field vectors, which linearly oscillate and circularly rotate in time for linear and circular polarizations, respectively. In the case of LG beams, the directions of the electric field vectors depend on the azimuthal angle. The grey shadings indicate the electric field intensity distribution. The white bar indicates a scale of 1 µm. (C) Near-field enhancement spectra of the plasmonic multimers (Aa–Ad) excited by the G and LG beams (Ba–Bd), respectively. The near-field spectra are obtained at a 1-nm distance from the top corner of a metallic vertex facing the gap; the intensities are normalized by the incident fields. (D) Instantaneous electric field distributions within the multimer gaps. The wavelengths of the incident beams are set at the resonant peaks of corresponding near-field spectra (Ca–Cd). Yellow arrows represent electric field vectors, and the grey shadings indicate the intensity distribution. The motions of the electric field vector are shown in Supplementary Movies S1–S6. The white bar indicates a scale of 1 nm.

We performed numerical simulations of the localized plasmonic fields within the nanogaps. The first model system is a dimer structure consisting of two triangular nanoparticles called a bowtie antenna (Figure 2Aa). Gold triangular blocks with 150-nm sides, 5-nm corner curvatures, and 30-nm thicknesses were placed 10 nm apart. The triangular dimer was excited by a normally illuminated G beam linearly polarized (LP) along the dimer axis (Figure 2Ba). Figure 2Ca shows the near-field spectrum obtained at a 1-nm distance from one of the corner tops facing the gap on the upper surface. The spectrum was normalized to the incident field intensity. An instantaneous electric field distribution in the dimer gap excited at the resonant peak of 1.5945 eV is shown in Figure 2Da (see Supplementary Video S1). The results show LP strong localized field at the gap center. When replacing the incident beam with CP light, the LP gap field exhibits no considerable changes (see Supplementary Video S2), indicating that the SAMs of photons are not transferred to gap plasmons of the dimer structure. Recently, it was reported that high-order multipolar plasmon modes of the dimer structure could be excited to form finely structured fields for extremely small gaps (∼1 nm) [25]. However, LP electric fields were observed within the gap along the dimer axis. Biagioni et al. proposed using a cross-dipole antenna, a plasmonic tetramer structure, to localize CP light in the nanogap [26]. However, the tetramer is not a necessary or sufficient condition for SAM coupling.

We propose a plasmonic trimer as the simplest structure to preserve circular polarization [27, 28]. In addition, the interaction between the trimer of the triangular blocks surrounding a nanogap (Figure 2Ab) and CP-G beam (Figure 2Bb) was numerically analyzed. The two-dimensional gap was defined as a circular area with a 10-nm diameter. The resonant peak is red-shifted to 1.5696 eV (Figure 2Cb) because the distance between neighboring triangle blocks is shorter than that of the dimer [20]. The gap-field distribution at the resonant peak shows SAM coupled with the plasmonic trimer and successfully confined to the nanogap (Figure 2Db, Supplementary Video S3). The field enhancement reaches 10^4^, and the trimer can form LP gap fields in any direction.

The degrees of freedom in the trimer structure are insufficient for receiving OAM. When LG beams (Figure 2Bc and Bd) are incident on the trimer, no intense localized field appears within the gap. Hereafter, the LG modes are indexed by the azimuthal mode number *l* and polarization helicity *s*, representing the wavefront of exp(i*lϕ*) (where *ϕ* is the azimuthal angle) and the handedness of circular polarizations (*s* = ±1), corresponding to the OAM and SAM of *lh* and *sh* per photon, respectively [1]. The radial mode number *p* also characterizes optical vortices; only the lowest-order mode of *p* = 0 is treated in the following simulation and discussion. The G beam in Figure 2Bb is the LG mode (*l* = 0, *s* = 1), whereas the LP-G-mode in Figure 2Ba is given as the interference between the two modes (*l* = 0, *s* = 1) and (*l* = 0, *s* = −1). The LG beams in Figure 2Bd and Bc are the mode (*l* = 1, *s* = 1) and superposition of the two modes (*l* = 1, *s* = 1) and (*l* = −1, *s* = −1), respectively.

To confine the optical vortex field into the nanospace, we originally designed a triangular tetramer (Figure 2Ac) and pentamer (Figure 2Ad) having quadrupolar and quintupolar antenna structures, respectively. When the plasmonic tetramer and pentamer with 10-nm-diameter gaps are excited by the normal illumination of LP-LG and CP-LG beams (Figure 2Bc and Bd), the near-field intensity spectra (Figure 2Cc and Cd) considerably enhances (>10^7^) the gap-mode localized plasmonic fields at resonant peaks of 1.4375 eV and 1.2915 eV, respectively. Their spectral shapes, including the enhancement factors and resonant peaks, remain the same even when the LP-LG and CP-LG beam illuminations are interchanged. Figure 2Dc shows that the tetramer structure formed an LP gap field with an azimuthally dependent polarization direction (see Supplementary Video S4). Furthermore, the CP electric field with an azimuthally variant phase shift was successfully localized in the pentamer gap, as shown in Figure 2Dd (see Supplementary Video S5). These results are the first demonstration of nanoscale shaping of vortex photons based on OAM and SAM transfer.

The tetramer gap formed the LP vortex field, even when illuminated with the CP-LG beam (see Supplementary Video S5). Namely, the SAM is rejected in the transfer process from photon to gap plasmon. In contrast, the pentamer gap can localize all LG modes in Figure 2Ba–Bd by conserving OAM and SAM. Table 1 summarizes the relationship between the triangular multimer structures and vortex fields formed within the nanogaps. These results indicate that the LP-LG modes given by the interference between modes (*l*, *s*) and (–*l*, –*s*) can be confined to multimer gaps under the condition *N* ≥ 2|*l* + *s*| (*N* is the number of triangular blocks). In addition, the OAM and SAM of CP vortex beams can be transferred to nanogap plasmons when *N* > 2|*l* + *s*|.

**Table 1:** Relation between some basic triangle multimer structures and the nano-vortex fields that can be formed within their nano-gaps. In the case of LP fields, the “+” sign connecting two modes with opposite spins represents the interference between the two modes that are simultaneously excited.

*N*	Achievable LP fields (l, s)	Achievable CP fields (l, s)
2	(0, +1) + (0, −1)	Unable
3	(0, +1) + (0, −1)	(0, +1), (0, −1)
4	(0, +1) + (0, −1), (+1, +1) + (−1, −1)	(0, +1), (0, −1)
5	(0, +1) + (0, −1), (+1, +1) + (−1, −1)	(0, +1), (0, −1) (+1, +1), (−1, −1)
6	(0, +1) + (0, −1), (+1, +1) + (−1, −1), (+2, +1) + (−2, −1)	(0, +1), (0, −1) (+1, +1), (−1, −1)

## 3 Discussion

**Figure 3:**
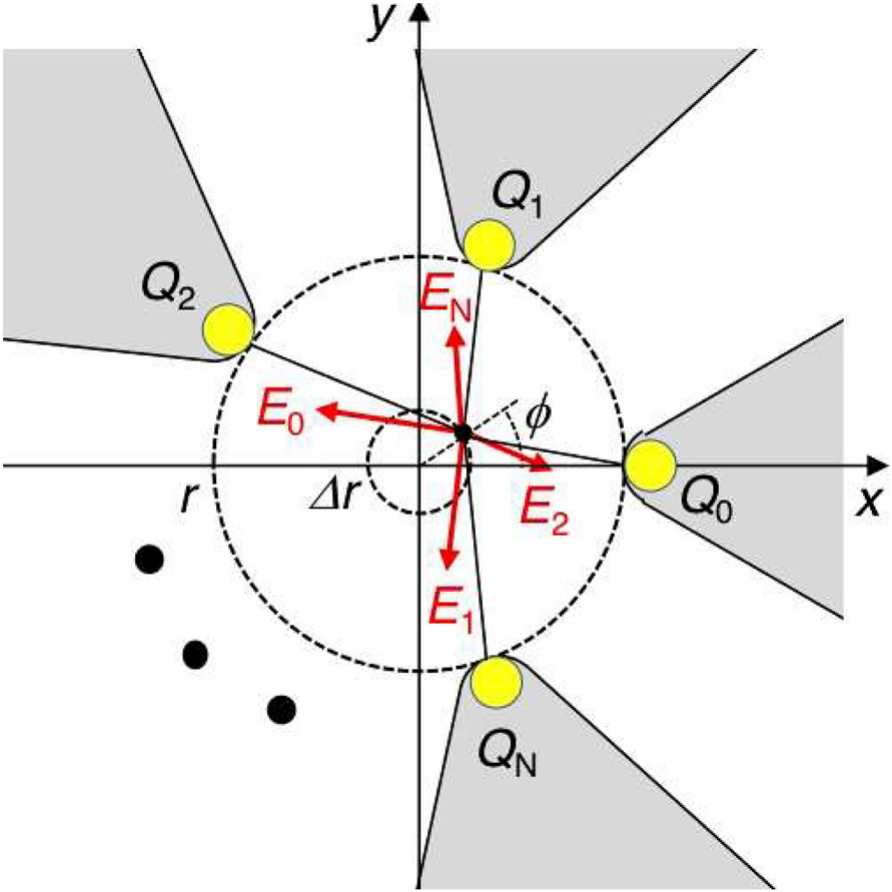
Electric field model created in a gap surrounded by *N* triangular metal blocks.

We theoretically verified the characteristic properties of plasmonic multimer gap fields. When a multimer consisting of *N* triangular metal blocks is excited by a CP-LG beam with mode (*l*, *s*), as shown in Figure 3, oscillating electric charges are densely generated at individual triangular apexes facing a nanogap. The induced charges at the *n*th triangle are given by

(1) $$Q_{n}\left(t\right)=-Q\cos\left\{2\pi \left(l+s\right)n/N+\omega t+\theta_{0}\right\}$$

where *ω* represents the angular frequency of the illumination light and *θ*_0_ denotes the phase delay between the collective electron oscillation and incident field. The electric field in the gap area is created by the point charges at *N* triangular apexes separated by distance *r* from the gap center (Figure 3). Accordingly, we calculated the localized field at the gap center, which is the near-field interference of the longitudinal electric waves generated by the point charges. The complex electric field *E*(Δ*r*, Φ) = *E*_*x*_(Δ*r*, Φ) + i*E*_*y*_(Δ*r*, Φ) (*E*_*x*_ and *E*_*y*_ are *x*- and *y*-components, respectively), evaluated at a distance Δ*r* from the gap center (origin of coordinates) and an azimuthal angle Φ from the *x*-axis*,* is expressed as

(2) $$E\left(\Delta r,\phi ,t\right)=\frac{1}{4\pi \varepsilon }\overset{N-1}{\underset{n=0}{\sum }}\frac{Q_{n}\left(t\right)\left\{\Delta r{\text{e}}^{i\phi }-re^{i2\pi n/N}\right\}}{{\left[{\left\{\Delta r\cos\left(\phi \right)-r\cos\left(2\pi n/N\right)\right\}}^{2}+{\left\{\Delta r\sin\left(\phi \right)-r\sin\left(2\pi n/N\right)\right\}}^{2}\right]}^{\frac{3}{2}}}$$

Substituting Eq. (1) and expressing the terms as an infinite series,

(3) $$\begin{array}{ll} E\left(\Delta r,\phi ,t\right) & =\frac{Q}{2\pi \varepsilon {\left(r^{2}+\Delta r^{2}\right)}^{\frac{3}{2}}}\overset{N-1}{\underset{n=0}{\sum }}\overset{\infty }{\underset{m=0}{\sum }}\overset{m}{\underset{k=0}{\sum }}{\left(\frac{r\Delta r}{r^{2}+\Delta r^{2}}\right)}^{m}\frac{\overset{m}{\underset{j=0}{\prod }}\left(j+\frac{1}{2}\right)}{k!\left(m-k\right)!}\left(r{\text{e}}^{\text{i}2\pi n/N}-\Delta r{\text{e}}^{\text{i}\phi }\right)\times \cos\left\{2\pi \left(l+s+2k-m\right)n/N\right.\\ & \;-\left.\left(2k-m\right)\phi +\omega t+\theta_{0}\right\} \end{array}$$

Eq. (3) can be regarded as a Fourier series expression or discrete Fourier transform. We assume the approximation of Δ*r* ^2^ ≪ *r* ^2^. Under the condition $N>2\left|l+s\right|$ $\left(l+s\neq 0\right)$ and considering only the lowest-power term of the polynomial ${\left(\Delta r/r\right)}^{m}$ , Eq. (3) is rewritten as

(4) $$\begin{array}{ll} E\left(\Delta r,\phi ,t\right) & =\frac{NQ}{2\pi \varepsilon r^{2}}{\left(\frac{\Delta r}{r}\right)}^{\left|l+s\right|-1}\frac{\overset{\left|l+s\right|-1}{\underset{j=0}{\prod }}\left(j+\frac{1}{2}\right)}{\left(\left|l+s\right|-1\right)!}\\ & \;\times {\text{e}}^{-\text{i}\left\{\left(\left|l+s\right|-1\right)\phi \right\}}{\text{e}}^{\mp \text{i}\left(\omega t+\theta_{0}\right)} \end{array}$$

where the minus–plus sign corresponds to the case of positive or negative total angular momentum (*l* + *s*), respectively. When *ls* ≥ 0 such as (*l* = 0, *s* = ±1), (*l* = 1, *s* = 1), and (*l* = −2, *s* = −1), the azimuthal angle dependence of the localized field (Eq. (4)) is the same as that of the LG-mode field illuminating the plasmonic multimer. Namely, the OAM and SAM of CP-LG-mode photons can be confined into the nanogap plasmons when the number of triangular blocks satisfies the condition $N>2\left|l+s\right|$ . This condition has an analogy with the sample-rate criterion of the Nyquist–Shannon sampling theorem. When $N=2\left|l+s\right|$ $\left(l+s\neq 0\right)$ , Eq. (3) is given as

(5) $$\begin{array}{ll} E\left(\Delta r,\phi ,t\right) & =\frac{NQ}{2\pi \varepsilon r^{2}}{\left(\frac{\Delta r}{r}\right)}^{\left|l+s\right|-1}\frac{\overset{\left|l+s\right|-1}{\underset{j=0}{\prod }}\left(j+\frac{1}{2}\right)}{\left(\left|l+s\right|-1\right)!}\\ & \;\times {\text{e}}^{-\text{i}\left\{\left(\left|l+s\right|-1\right)\phi \right\}}\cos\left(\omega t+\theta_{0}\right) \end{array}$$

This equation expresses the LP field corresponding to the interference of the LG modes (*l*, *s*) and (–*l*, –*s*). Thus, the OAM of LP-LG-mode photons can be confined into multimer gaps when $N\geq 2\left|l+s\right|$ . These theoretical analyses can explain the simulation results in Figure 2.

**Figure 4:**
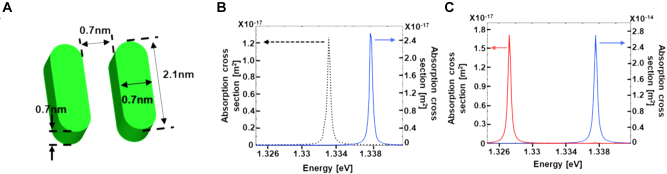
Interaction between nano-vortex fields and a dimer molecule. (A) Numerical model of the dimer molecule. (B, C) Absorption cross section spectra. (B) Molecule without plasmonic nanostructures with CP-G beam illumination. The broken and solid curves are the spectrum of monomer and dimer molecules, respectively. (C) Dimer molecule that is placed at the center of the nano-gap in the triangle pentamer. The blue and red curves are the spectra of the G beam and LG-mode excitation, respectively.

We also numerically simulated the interaction between the nano-vortex fields and a molecule to demonstrate the possibility of breaking down the selection rules for molecular transitions. The model was a dimer composed of two nanorod monomers as shown in Figure 4A. The electric susceptibility of the nanorods is given by

(6) $$\chi =\frac{{\left|d\right|}^{2}}{\hbar V}\frac{1}{\omega_{0}-\omega -i\gamma }$$

where *d* and *V* are the dipole moment and volume of the monomer, respectively; *ω*_0_ is the angular frequency of the monomer resonant state; *γ* is the nonradiative damping constant. The dipole moment *d* and damping parameter ℏγ were set to 8 D and 0.2 meV, respectively, near the value of porphyrin-based organic molecules [29, 30]. Because of the minimum mesh size limitation, the volume of the molecule was set to 0.96 nm^3^. We selected a monomer resonant frequency *ℏω*_0_ = 1.3332 eV to match the resonant frequency of the multimer structure. Because of the interaction between the two monomers (dipole–dipole coupling), two split states are formed, and their resonant frequencies are higher and lower than those of the non-coupled monomer. In the higher-frequency state, the dipoles of the respective monomers oscillate in phase. This state can be excited by a normally incident plane wave, corresponding to an OA state. In contrast, the lower-frequency state is the OF state, where the dipoles of the individual monomers exhibit anti-phase oscillations. This state cannot be excited by a normally incident plane wave, corresponding to an OF state.

First, a dimer molecule without plasmonic nanostructures was excited by the CP-G beam. The absorption cross section spectrum, calculated by dividing the volume integral of energy loss in the molecule by the intensity of incident light, is shown in Figure 4B. As a reference, the broken curve shows the absorption cross section spectra of a monomer under the same illumination conditions. The resonant frequency of the dimer (1.3381 eV) is blue-shifted from the monomer peak (1.3332 eV), which corresponds to the OA state. As expected, the lower-frequency peak of the OF state did not appear. Then, the dimer molecule is placed at the center of the nanogap in the triangular pentamer and excited by normal illumination of the CP-G-mode (*l* = 0, *s* = 1) and LG mode (*l* = 1, *s* = 1) beams. The near-field intensity spectra evaluated near the molecule are shown in Figure 4C. The spectrum under G beam illumination (blue curve) has a peak at 1.3378 eV, which is the higher frequency OA state. The peak intensity is 1.1 × 10^3^ times higher than that without the plasmonic structure. In contrast, the LG mode excitation (red curve) exhibits a resonant peak at a lower frequency of the OF transition (1.3273 eV), demonstrating that the quadrupolar transition to the OF state of the dimer molecule can be excited by the nano-vortex field. Furthermore, the red curve has no peak at the OA state frequency, indicating that the OA transition is no longer OA under the LG-mode excitation owing to the conservation of angular momentum. The selective excitation of the OA and OF transitions can be controlled by the nanoscale shaping of the plasmonic field. This new possibility implies a complete breakdown of the selection rules. The OF transition can also be excited by the LG-mode beam without the pentamer structure, despite the negligibly low excitation efficiency. The simulation results show that the near-field intensity at the lower frequency peak, which corresponds to the OF transition rate, is enhanced 1.8 × 10^6^ times by a plasmonic pentamer. In other words, the OAM can be efficiently transferred from propagating vortex photons to molecules through nanogap plasmons.

Further enhancement can be achieved by sharpening the corners of the triangular structures and reducing the size of the nanogaps. In the above simulation, the corner curvature and gap diameter were set to 5 nm and 10 nm, respectively, which are realistic values for our fabrication accuracy [31]. The numerical simulation showed that the intensity enhancement of the 1-nm gap structure is four orders higher than that of the 10-nm gap structure, thus, the enhancement of the OF transition rate will reach 10^10^ when realizing a 1-nm gap structure. However, the quantum mechanical phenomena, including electron tunneling and nonlocal electrodynamic effects, will appear for gaps smaller than 1 nm [32, 33]. When the position of the molecule is shifted from the gap center, the quadrupolar transition and in-phase dipolar oscillation appear slightly under nano-vortex field excitation. Furthermore, the helically structured gap field is partially deformed if there is a misalignment between the optical axis of the LG beam illumination and the multimer structure center. The deformation is because the off-axis LG-mode beam is a linear combination of on-axis LG-mode beams with various (*l*, *s*), as they form an orthogonal base. Individual LG-mode beams form structured gap fields with various angular momenta, according to the relation mentioned above.

## 4 Conclusions

In conclusion, we designed plasmonic multimer structures that enable the forming of single-nanometer-scale optical vortices. By resonantly exciting the multimer structure, the OAM and SAM of the incident LG beam were transferred to a multipolar plasmonic mode and localized into a nanogap. We also numerically demonstrated the efficient and selective excitation of the OF state in a molecule placed in a nano-vortex field. Photochemical reactions and photophysical processes can be independent of the selection rules of electronic transitions by controlling the nano-shape of photons. In addition, we developed techniques to place and fix molecules within metal nanogaps by plasmon-assisted two-photon polymerization [34] and trapping and deposition with plasmonic optical forces [31, 35]. The OAM transfer from nano-vortex photons to molecules also induces a rotational optical force, that is, optical torque, which may lead to a nano-vortex flow of molecules and chiral structuring of molecular assemblies [36]. The optical nanoscale shaping method can open new research fields in photonic and material sciences.

## Supplementary Material

Supplementary Material Details
