# Supplementary material for: Nano-shaping of chiral photons
Source: Nanophotonics. 2023 May 16;12(13):2499–506. doi: 10.1515/nanoph-2022-0779 (PMC11502058; doi:10.1515/nanoph-2022-0779)
Supplement: Supplementary file 1 — Supplementary Material Details [file j_nanoph-2022-0779_suppl_001.docx]

Supplementary Materials

**Nano-Shaping of Chiral Photons**

Yuji Sunaba, Masaki Ide, Ryo Takei, Kyosuke Sakai, Christophe Pin, and Keiji Sasaki†,

Research Institute for Electronic Science, Hokkaido University, Sapporo 001-0020, Japan

†Corresponding author. E-mail: sasaki@es.hokudai.ac.jp

**Supplementary Note 1: Method of numerical simulation**

To investigate the plasmonic nanogap fields formed in the multimer nanostructures, we numerically calculated the electric fields in the systems using the finite element method in COMSOL Multiphysics. The simulation models are multimer nanostructures consisting of triangular gold blocks in air surrounded by a perfectly matched layer (PML) on the side and lower boundaries, and the scattering boundary condition on the top surface. The optical constant for gold was obtained from Johnson and Christy (*31*). We set the focused incident beam at the upper surface using the three components of the complex electric field (*E*_x_, *E*_y_, *E*_z_) given by vector diffraction theory (*32, 33*). The illumination beam propagated downwards, forming a beam waist on the gold nanostructures. We calculated the near-field intensity spectra to discuss the excitation of plasmonic resonances. The time-averaged intensity spectra were obtained at 1 nm from the top of one corner facing the gap at the top surface. The calculated electric field intensity (|*E*|^2^) was normalized by the field (|*E*_0_|^2^) obtained without the nanostructures.

**References**

[1] P. B. Johnson and R. W. Christy, “Optical constants of the noble metals,” *Phys. Rev. B,* vol. 6, no. 12. pp. 4370–4379, 1972. https://doi.org/10.1103/PhysRevB.6.4370

[2] B. Richards and E. Wolf, “Electromagnetic diffraction in optical systems, II. Structure of the image field in an aplanatic system,” *Proc. R. Soc. London, Ser. A,* vol. 253, no. 1274, pp. 358–379, 1959. https://doi.org/10.1098/rspa.1959.0200

[3] K. Kitamura, K. Sakai, N. Takayama, M. Nishimoto, and S. Noda, “Focusing properties of vector vortex beams emitted by photonic-crystal lasers,” *Opt. Lett.* vol. 37, no. 12, pp. 2421-2423, 2012. https://doi.org/10.1364/OL.37.002421

**Supplementary Note 2:** **Electric-field distribution in the gap of the multimer nanoantennas**


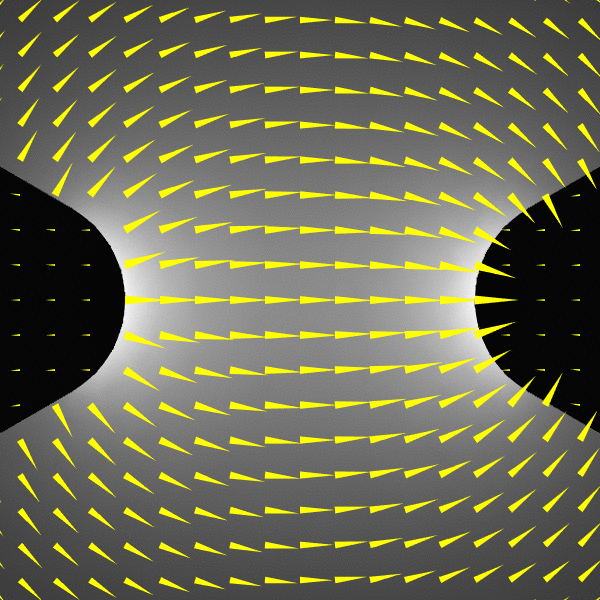


Video S1. Electric field distribution in the dimer gap excited by the normal illumination of a G beam is linearly polarized along the dimer axis.


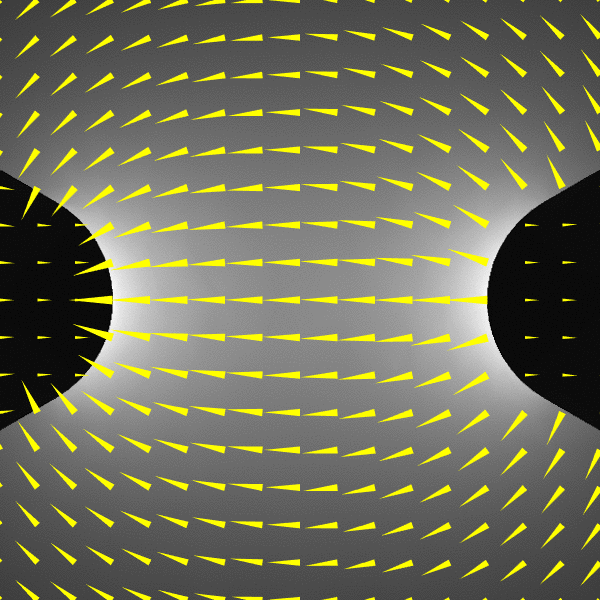


Video S2. Electric-field distribution in the dimer gap excited by CP light


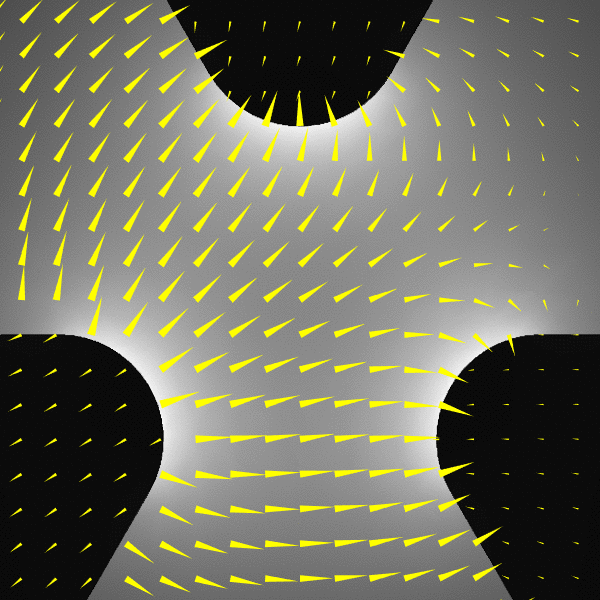


Video S3. Electric-field distribution in the trimer gap excited by CP light


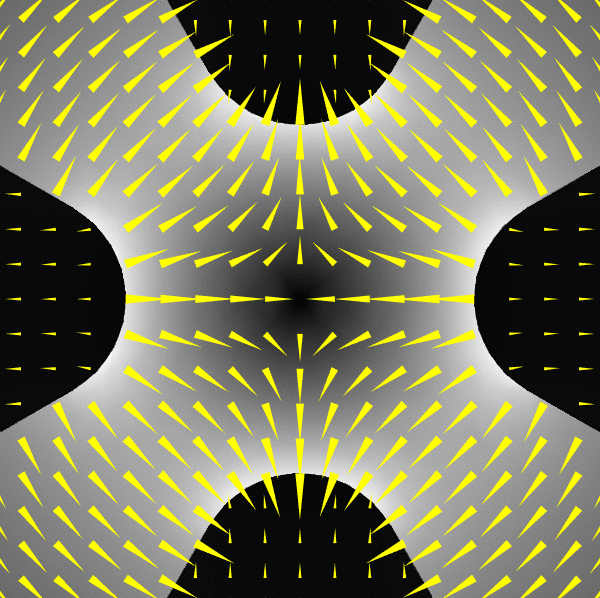


Video S4. Electric-field distribution in the tetramer gap excited by the LP-LG beam


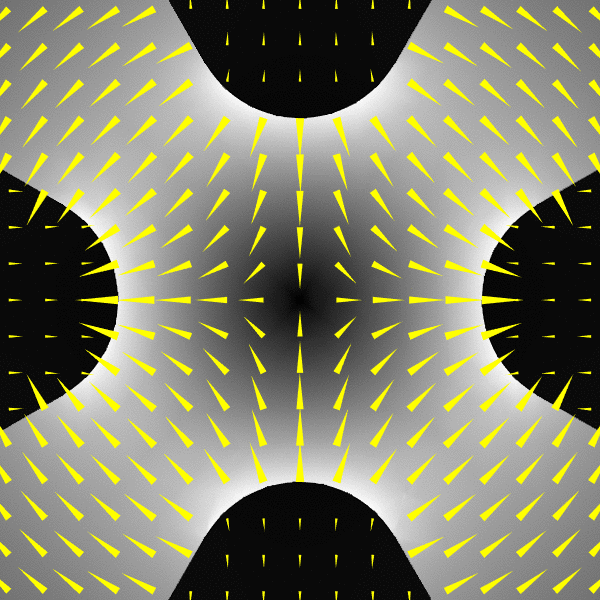


Video S5. Electric-field distribution in the tetramer gap excited by the CP-LG beam


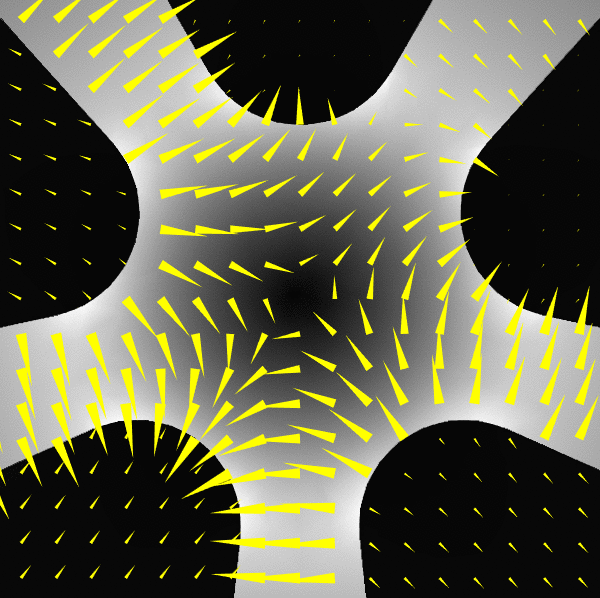


Video S6. Electric-field distribution in the pentamer gap excited by the CP-LG beam


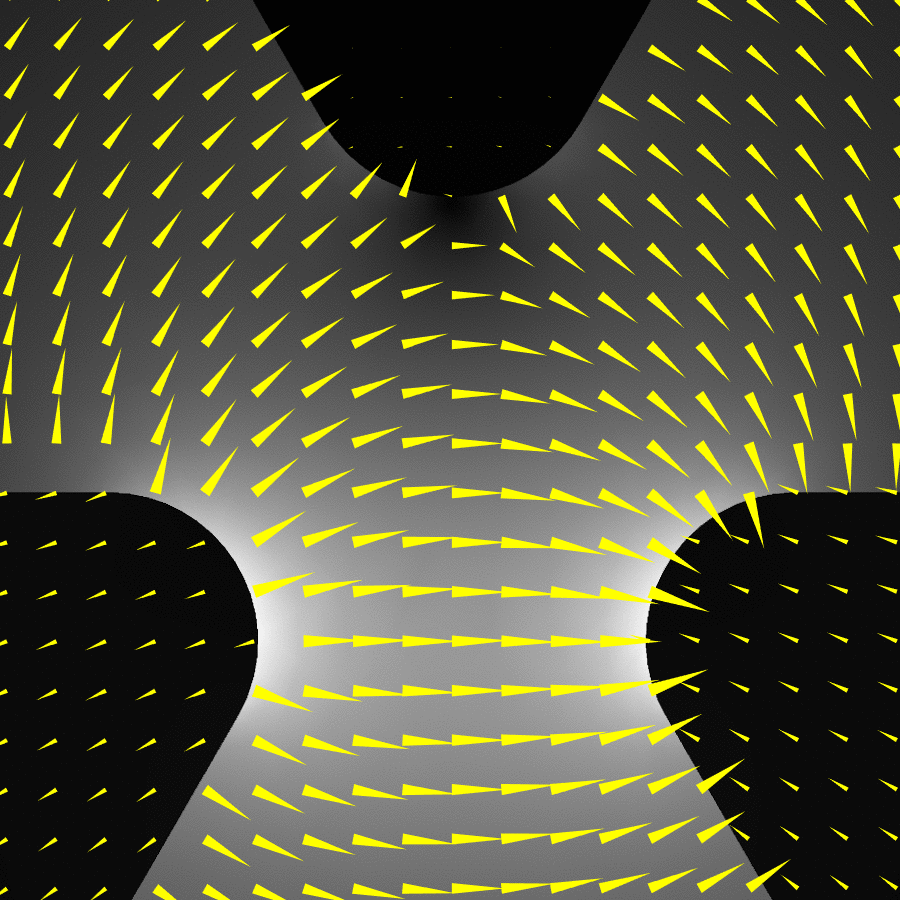


Video S7. Electric-field distribution in the trimer gap excited by the LP-G beam


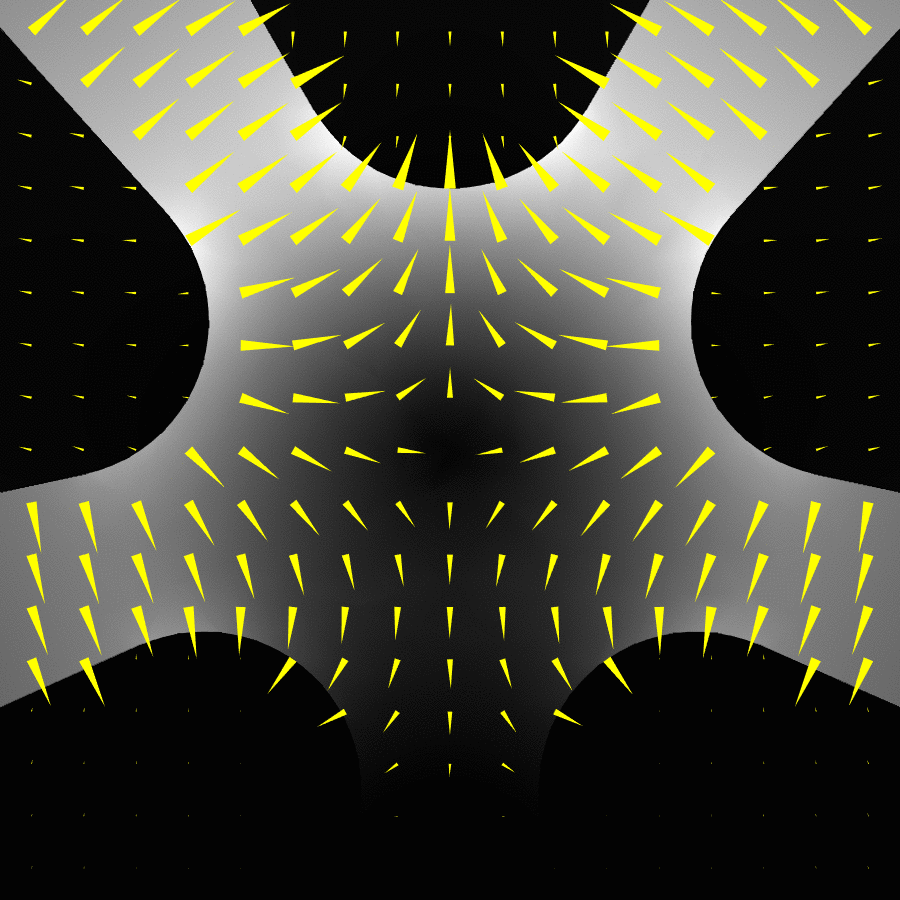


Video S8. Electric-field distribution in the pentamer gap excited by the LP-LG beam
